# Factors associated with the introduction of prelacteal feeds in Nepal: findings from the Nepal Demographic and Health Survey 2011

**DOI:** 10.1186/1746-4358-8-9

**Published:** 2013-08-08

**Authors:** Vishnu Khanal, Mandira Adhikari, Kay Sauer, Yun Zhao

**Affiliations:** 1 School of Public Health, Curtin University, Perth, WA, Australia; 2 Women’s Health Program, Population Services International, Nawalparasi, Nepal; 3 Centre for Behavioural Research in Cancer Control, Curtin University, Perth, WA, Australia

**Keywords:** Cross-sectional survey, Exclusive breastfeeding, Nepal, Prelacteal feeds

## Abstract

**Background:**

A prelacteal feed is any food except mother’s milk provided to a newborn before initiating breastfeeding. Prelacteal feeding is a major barrier to exclusive breastfeeding. It is a prevalent practice in Nepal. Little is known about the factors associated with providing prelacteal feeds to the Nepalese newborn. This study explored the factors associated with providing prelacteal feeds to children under three years in Nepal using the Nepal Demographic and Health Survey (NDHS) 2011.

**Methods:**

This study utilised the NDHS 2011 child dataset which is a nationally representative study. The rates of providing prelacteal feeds were reported as a proportion. Complex Sample Analysis method was used to account for the cluster design and sample weight of the study. Chi-square tests and multiple logistic regression were used to analyse the factors associated with providing prelacteal feeds.

**Results:**

A sample of 3948 mothers were included in the study. A total of 841 [26.5% (95% CI: 23.1%–30.3%)] weighted proportion) of mothers reported of providing prelacteal feeds to their newborn infants. Plain water (n = 75), sugar/glucose (n = 35), gripe water (n = 3), sugar/salt solution (n = 3), fruit juice (n = 3), infant formula (n = 96), tea (n = 3) and other milk other than breast milk (n = 556) were some of the types of prelacteal feeds reported. The multiple regression analysis showed that the mothers who had no education, were not working, were from the middle wealth quintile, who had not attended four antenatal care visits, were first time mothers and who were from the Terai/Plain region were more likely to provide prelacteal feeds.

**Conclusions:**

Given that one in four infants were provided with prelacteal feeds, there is a need to implement breastfeeding promotion programs to increase the practice of exclusive breastfeeding and reduce prelacteal feeding practices. Breastfeeding counseling at antenatal clinics and peer support for exclusive breastfeeding should be included as part of breastfeeding promotion programs. Mobilisation of female community health volunteers for peer counseling is also a feasible option for Nepal.

## Background

Exclusive breastfeeding (EBF) for first six months of life is beneficial to child and mother [1,2]. EBF protects a child from under nutrition and gastroenteristis. In the long run, EBF is protective against a number of chronic disease such as leukaemia, type 2 diabetes, and obesity [3,4]. Breastfeeding also has proven benefits for the neurocognitive development of the child, protection from childhood respiratory illness and protection for the mother from breast cancer. EBF also saves the cost associated with illnesses that arise out of the above mentioned illnesses [5]. For these reasons, the World Health Organization (WHO), United Nations Children’s Fund (UNICEF) and Ministry of Health and Population Nepal recommend and promote exclusive breastfeeding for first six months of life and continuation of breastfeeding thereafter [6-8].

Any food provided to a newborn before the initiation of mother’s breastfeeding is considered to be a prelacteal feed. The type of prelacteal feeds depends on the culture. It may include ghee (refined butter), honey, sugar, sugar juice, unboiled cow/goat milk etc [9]. The practice of prelacteal feeding is a major cultural practice still prevalent in many places throughout South Asia regions [9-12]. All prelacteal feeds are provided for non nutritional reasons such as clearing the throat/bowel; or thinking that mother’s milk is insufficient or the colostrum is too heavy for the newborn to digest [9]. Prelacteal feeds have lesser nutrient and immunological value; and are often likely to introduce contaminants [9].

Introduction of prelacteal feeds is a known barrier to continuation of exclusive breastfeeding. By definition, a child provided with prelacteal feeds is not exclusively breastfed. Knowledge on the determinants of introduction of prelacteal feeds is essential to promote exclusive breastfeeding and early initiation of breastfeeding [13,14]. To date, only one study from Nepal has reported on the determinants of prelacteal feeds [15]. That study had a small sample size and was confined to few village development committees (10 of 77) from one of the 75 districts in Nepal. A larger representative study covering the entire country is essential in Nepal so that findings can be generalized to the entire country. This study aimed to identify the determinants of prelacteal feeds amongst young children aged under three years using data from the Nepal Demographic Health Survey (NDHS) 2011.

## Methods

The child dataset of NDHS 2011 [16], a nationally representative cross-sectional survey based on multi stage cluster sampling was used. In the first stage of the sampling, the enumeration areas (EA) were selected. A total of 95 urban and 194 rural EAs were included in the survey. In stage two, households were selected based on the EAs. The NDHS utilises three sets of validated questionnaires to collect data and includes a household questionnaire, a mother’s questionnaire and a men’s (father’s) questionnaire. The child dataset used in this study included all the relevant information on child health from all of three questionnaires. The current NDHS 2011 has a response rate of 95.3%. Details of sampling technique, selection of households, questionnaire and validation procedure are published in the survey report [16].

### Statistical analysis

The prevalence of providing prelacteal feeds were reported as a proportion. The association between the categorical independent variables of interest with outcome variable (prelacteal feeds) were tested by using a chi-square test (*χ*^2^). The significant varaibles (p < 0.05) were then analysed by using multiple logistic regression methods. We used a Complex Sample Analysis Procedure which accounted for the cluster sampling design and sample weight to provide generalisable and accurate estimates of proportion, probability values and odds ratios [17-19]. A p-value <0.05 was considered statistically significant. Statistical analysis was performed by using IBM SPSS Statistics for Windows, Version 20.0 [20].

Given that the NDHS included a number of socio-demographic variables, the interaction of such variables is likely. Therefore, we examined the correlation between some overlapping independent variables. Ecological regions had an interaction with the development regions (both divide the country geographically). Likewise, birth order and birth interval had an interaction. Therefore, in the initial model (model 1) we excluded the development region and birth order to avoid such interactions. In next step (model 2), the ecological region was replaced by development region; and birth interval was replaced by birth order.

The current data anlaysis protocol was approved from Curtin University Human Research Ethics Committee [protocol approval-SPH-16-2012]. The DHS was approved by Nepal Health Research Council, Nepal.

### Definition of variables

#### Outcome variable

The outcome variable was prelacteal feeds. The NDHS asked the mother two questions to assess if the child was provided with any prelacteal feeds [16]; In the first three days after delivery, was (NAME of the child) given anything to drink other than breast milk? What was (NAME of the child) given to drink? [Options were : milk (other than breast milk); plain water; sugar or glucose water, gripe water, sugar salt water solution; fruit juice; infant formula; tea infusion; coffee, honey; and/others]. Prelacteal feeds; NDHS counted prelacteal feeds provided within the first three days before mothers milk [16]. The main outcome variable (provided prelacteal feeds = 1 and did not provide prelacteal feeds = 0) was derived from the response to the first question. The types of prelacteal feeds were reported as a frequency.

#### Independent variables

Four major types of variables: socio-demographic factors (ethnicity, religion and wealth quintile), maternal factors (mother’s age at pregnancy, maternal education, maternal occupation, antenatal visit and place of delivery), child related factors (sex of the child, birth order, birth interval, and size of baby at birth) and contextual factors (place of residence, development regions and ecological regions) were included in the study based on the literature review and the available data in the NDHS dataset. Ethnicity was based on the caste system in Nepal and it was divided into three major groups based on available literature and similarities between the caste/ethnic groups (i) advantaged (ii) disadvantaged (Janjati) (iii) disadvantaged (Dalit) [15,21]. The advantaged group included Brahmin, Chhetri, Thakuri and Sanyasi. The disadvantaged Janjati included indigenous groups. Disadvantaged Dalit included the castes that were considered lower in the caste based stratification within Nepalese society according to *Manusmriti*[15,21]. The religions recorded in the survey were Hindu, Buddhist, Muslim, Kirat and Christian. Given the small number of non-Hindu respondents, religion was recoded into (i) Hindu and (ii) Others. The wealth quintile is a composite indicator that divides households into five categories (i) poorest (ii) poor (iii) average (iv) rich and (v) richest. The wealth quintile uses principle component analysis based on more than 40 assets variables [16] and this was then recoded into three categories; the lowest 40% (poorest and poor) as poor; middle 40% (average and rich) as average; and the upper 20% (richest) as rich [22].

Mother’s age at pregnancy was derived by subtracting the age of child and nine months from the mother’s current age. It is then recoded into 15–19 years, 20–29 years, 30–34 years, and > =35 years. Maternal occupation was recoded as not working, working in agriculture, and working (paid work). Maternal education was recorded as no education, primary, secondary, and higher. The number of antenatal clinic (ANC) visits was recorded as a continuous variable and was also categorized into no ANC visits, one to three ANC visits, and four or more ANC visits (recommended number of visits). The size of baby was based on the mother’s perception and recorded as very small, smaller than average, average, larger than average, and very large. It was later re-categorized into small (very small and smaller than average), average, and large (larger than average and very large). Places of delivery included hospital (public or private), primary health care centres, health posts, sub health posts, and private clinics. These were classified as a health facility and the rest were classified as a home birth. Birth interval or spacing of the child was re-coded into three categories (i) no previous birth; (ii) less than 24 months and (ii) 24 months or more. Birth order was recorded as a continuous variable which was then recoded into three categories; (i) first (ii) second or third, and (iii) fourth or more.

Nepal is divided into five vertical geographical sections for administrative purposes (the North to South) which includes; Eastern development region, Central development region, Western development region, Mid-western development region and Far-western development region. Ecological zones divide the country based on the altitude and the climate. The Mountain (the North part covered with snow most of the time), the Hills (the middle part with mountains but not covered with the snow most of time of year) and the Terai (plains area along the border of India ranging from the East to West with a hotter climate most of the time of the year). The ecological region and development regions overlap being the vertical and horizontal sections of country. Each ecological region contains all five development regions and each development region has all three ecological regions. In the Nepalese community, the cultural practices vary based on their ethnicity, development regions and ecological regions.

## Results

### Characteristics of respondents

Table 1 reports the characteristics of 3948 respondents. The majority (64.2%) of mothers were in the age group 20–29 years. Less than a half (43.1%) did not have any formal education and 61.2% were working in the agriculture sector. Slightly more than half (53.1%) had attended four or more antenatal care visits during their last pregnancy and the majority (60.2%) had their child at home. More than half of the mothers were from disadvantaged ethnic groups [Janjati (34.6%) and Dalit (17.5%)]. Almost half (48.7%) were from poor families. About one third (30.6%) of the mothers were first time mothers; and the majority (56.6%) of the mothers had a birth interval of ≥24 months. About one in five (17.5%) mothers perceived that their child was smaller than average. The majority (78.1%) were from rural area. A total of 17.7% were from the Far western development region and 41.4% were from Terai/Plain area.

**Table 1:** Rate (%) of providing prelacteal feeds among children by demographic and socioeconomic characteristics, Nepal 2011 (n = 3948)

**Factor**	**Total n (%) #**	**Provided prelacteal feeds**	**P-value**
**Maternal Factors**			
**Mother’s age at pregnancy**			**0.263**
15–19	292 (7.4)	41 (22.4)	
20–29	2534 (64.2)	330 (24.0)	
30–34	641 (16.2)	38 (19.7)	
≥35	481 (12.2)	42 (30.7)	
Maternal Education			**<0.001**
No education	1701 (43.1)	380 (31.1)	
Primary	786 (19.9)	117 (17.9)	
Secondary	1195 (30.3)	239 (22.2)	
Higher	266 (6.7)	105 (42.6)	
**Mother’s occupation**			**<0.001**
Not working	938 (23.8)	301 (37.1)	
Agriculture	2416 (61.2)	416 (21.7)	
Working (paid)	594 (15.0)	124 (25.1)	
**ANC visit (Times)**			**0.009**
No ANC visit	582 (14.7)	112 (25.2)	
1–3	1271 (32.2)	306 (31.9)	
4 or more	2095 (53.1)	423 (23.1)	
**Place of delivery**			**0.152**
Home	2378 (60.2)	488 (27.8)	
Health facility	1570 (39.8)	353 (24.5)	
Sociodemographic factors			
*Ethnicity*			**0.320**
Advantaged	1890 (47.9)	138 (29.1)	
Disadvantaged (Janjati)	1366 (34.6)	274 (24.1)	
Disadvantaged (Dalit)	692 (17.5)	129 (25.4)	
*Religion*			**0.817**
Hindu	3369(85.3)	710 (26.3)	
Others	579 (14.7)	131 (27.6)	
*Wealth quintile*			**0.002**
Poor (Lower 40%)	1922 (48.7)	315 (21.2)	
Middle (Middle 40%)	1373 (34.8)	334 (31.3)	
Rich (Upper 20%)	653 (16.5)	192 (29.9)	
**Child related factors**			
*Sex of child*			**0.019**
Male	2116 (53.6)	416 (24.6)	
Female	1832 (46.4)	425 (28.6)	
*Birth order*			**0.001**
First	1209 (30.6)	329 (31.7)	
Second or third	1797 (45.5)	322 (22.3)	
Fourth or more	942 (23.9)	190 (27.7)	
*Birth interval*			**0.035**
No previous birth	1214 (30.7)	333 (32.0)	
<24 months	500 (12.7)	82 (24.0)	
≥24 months	2234 (56.6)	426 (23.9)	
*Size of baby*			**0.011**
Average	2499 (63.3)	547 (27.9)	
Small	960 (17.5)	160 (28.1)	
Large	756 (19.2)	133 (20.2)	
**Contextual Factors**			
*Place of residence*			**0.622**
Urban	864 (21.9)	200 (27.8)	
Rural	3084 (78.1)	641 (36.4)	
*Development region*			**<0.001**
Eastern	925 (23.4)	182 (21.2)	
Central	831 (21.0)	274 (40.7)	
Western	627 (15.9)	169 (26.4)	
Mid-Western	865 (21.9)	168 (19.8)	
Far-Western	700 (17.7)	48 (7.1)	
*Ecological region*			**<0.001**
Mountain	713 (18.1)	108 (15.6)	
Hill	1602 (40.6)	256 (17.3)	
Terai/Plain	1633 (41.4)	477 (35.0)	

The percentages presented for the prelacteal feeds are the weighted and cluster sampling adjusted percentage which differs from the crude percentage. The number of missing values may vary for each variable. # the number and percent reported are unweighted for the independent variables and their sub class to facilitate the reading as weighted count (frequency) will be in decimal points after accounting for sample weight and design effect.

### Prevalence of introduction of prelacteal feeds

Of 3948 children, 841 [21.3% unweighted proportion; and 26.5% (95% CI: 23.1, 30.3%) weighted proportion] were provided with prelacteal feeds. The type of prelacteal feeds included plain water (n = 75), sugar/glucose solution (n = 35), gripe water (n = 3), sugar/salt solution (n = 3), fruit juice (n = 3), infant formula (n = 96), tea (n = 3), other milk other than breastmilk (n = 556) and the rest were not specified.

### Factor associated with introduction of prelacteal feeds

Results of the chi- square tests to determine the factors associated with providing prelacteal feeds are shown in Table 1. The wealth quintile, age of mother at pregnancy, maternal education, mother’s occupation, the number of ANC visits, sex of child, birth order, birth interval, size of child at birth, ecological region and development region were significant (p < 0.05) variables. Table 2 shows the multiple logistic regression analysis. In model 1, maternal education, wealth quintile, mother’s occupation, ANC visits, birth interval and ecological region were significantly associated with providing prelacteal feeds. When compared to the mothers with no education, mothers with primary (OR 0.45; 95% CI 0.34, 0.60) and secondary education (OR 0.53; 95% CI (0.39, 0.73) were less likely to provide prelacteal feeds. The mothers from the middle wealth quintile (OR 1.45; 95% CI 1.05, 1.99), mothers who were not working (OR 1.49; 95% CI 1.06, 2.08), mothers who did not attend any ANC visits (OR 1.65; 95% CI 1.08, 2.52) or attended 1–3 ANC visits (OR 1.71; 95% CI 1.32, 2.21); first time mothers with no previous birth (OR 1.71; 95% CI 1.38, 2.12), and the mothers from the Plain/Terai region (OR 2.28; 95% CI 1.46, 3.57) were more likely to provide their children with prelacteal feeds than their counterparts (Table 2).

**Table 2:** Factors associated with providing prelacteal feeds in Nepal 2011-adjusted and unadjusted odds ratio

**Factor**	**Crude OR (95% CI)**	**Adjusted OR (95% CI) Model 1**	**Adjusted OR (95% CI) Model 2**
**Maternal education**		p < 0.001	p < 0.001
No education	1.00	1.00	1.00
Primary	0.48 (0.35, 0.66) *	0.45 (0.34, 0.60) *	0.45 (0.34, 0.59)*
Secondary	0.63 (0.47, 0.84) *	0.53 (0.39, 0.73) *	0.54 (0.40, 0.73)*
Higher	1.64 (1.07, 2.54) *	1.54 (0.92, 2.85)	1.33 (0.80, 2.20)
**Wealth quintile**		p = 0.057	p = 0.011
Poor (lower 40%)	1.00	1.00	1.00
Middle (middle 40%)	1.69 (1.21, 2.35) *	1.45 (1.05, 1.99)*	1.63 (1.18, 2.26)*
Rich (top 20%)	1.58 (1.11, 2.25) *	1.35 (0.87, 2.09)	1.39 (0.90, 2.14)
**Mother’s occupation**		p = 0.006	p = 0.004
Not working	1.76 (1.23, 2.53) *	1.49 (1.06, 2.08)*	1.43 (1.06, 2.03)*
Agriculture	0.82 (0.61, 1.11)	0.99 (0.72, 1.37)	0.96 (0.70, 1.33)
Working (paid)	1.00	1.00	1.00
**ANC visit (number)**		p < 0.001	p = 0.001
No ANC visit	1.12 (0.72, 1.72)	1.65 (1.08, 2.52)*	1.23 (0.81, 1.87)*
1–3	1.55 (1.20, 2.02) *	1.71 (1.32, 2.21)*	1.62 (1.24, 2.10)*
4 or more	1.00	1.00	1.00
**Birth interval**		p < 0.001	Not in model
No previous birth	1.49 (1.18, 1.90) *	1.71 (1.38, 2.12) *	
< 24 months	1.00 (0.56, 1.69)	0.92 (0.57, 1.46)	
> = 24 months	1.00	1.00	
**Sex of child**		p = 0.062	p = 0.08
Male	1.00	1.00	
Female	1.22 (1.03, 1.44) *	1.17 (0.99, 1.39)	1.17 (0.98-1.39)
**Ecological region**		p < 0.001	Not in model
Mountain	1.00	1.00	
Hill	1.13 (0.73, 1.75)	1.10 (0.70, 1.73)	
Terai/Plain	2.90 (1.86, 4.54) *	2.28 (1.46, 3.57) *	
**Size of baby**		p = 0.093	p = 0.038
Average	1.00	1.00	1.00
Small	1.01 (0.76, 1.33)	1.11 (0.83, 1.49)	1.24 (0.94, 1.64)
Large	0.65 (0.49, 0.87) *	0.75 (0.57, 1.01)	0.77 (0.58, 1.02)
**Development region** ®		Not in model	p < 0.001
Eastern	3.51 (2.16, 5.72) *		3.15 (1.94, 5.11)*
Central	8.99 (5.32, 15.19) *		6.84 (4.14, 11.32)*
Western	4.71 (2.80, 7.93) *		4.54 (2.56, 7.97)*
Mid-Western	3.07 (1.84, 5.12) *		2.84 (1.70, 4.75)*
Far-Western	1.00		1.00
**Birth order** ®		Not in model	p < 0.001
First	1.00		1.00
Second or third	0.62 (0.49, 0.78) *		0.59 (0.48, 0.73)*
Fourth or more	0.82 (0.59, 1.14)		0.72 (0.55, 0.96)*

*Statistically significant CI.

Independent variables entered in the initial model: Model 1: maternal education, wealth quintile, mother’s occupation, ANC visit, sex of the child, birth interval, child’s size at birth, and ecological region. [d.f.16 Wald chi square value:184.397 (p < 0.001); Hosmer-Lemeshow goodness of fit test: 0.431].

Model 2: maternal education, wealth quintile, mother’s occupation, ANC visit, sex of the child, birth order, child’s size at birth, and development region. [d.f. 18; Wald chi square value; 196.279 (p < 0.001); Hosmer-Lemeshow goodness of fit test: 0.998]. ® in model 2: Ecological zone was replaced by development region and the birth interval was replaced by birth order in the initial model.

There was a significant interaction of birth order and birth interval; and ecological region and development region (p < 0.001). Therefore, a second model (model 2) was developed which excluded birth order and development region from model 1 and entered birth order and the development region into the new model 2. While other significant independent variables of model 1 remained significant, development region and birth order also were significant in new model. The mothers from the Eastern region (OR 3.15; 95% CI 1.94, 5.11), Central region (OR 6.84; 95% CI 4.14, 11.32), Western region (OR 4.54; 95% CI 2.56, 7.97) and Mid-western region (OR 2.84; 95% CI 1.70-4.75) were more likely to provide prelacteal feeds to their newborns. The mothers whose child was the second or third child (OR 0.59; 95% CI 0.48, 0.73), or fourth or more (OR 0.72; 95% CI 0.55, 0.96) birth order were less likely to provide prelacteal (Table 2).

## Discussion

Benefits of initiation of breastfeeding within one hour of birth, exclusive breastfeeding for six months and the continuation of breastfeeding along with complementary feeding after six months have been well documented and meet international guidelines [1,6,7,23,24]. However, in some cultures including Nepal there is a preference for the introduction of prelacteal feeds [12,14,15,25]. This study reported a prevalence of prelacteal feeding (21.3%) which is lower than two previous Nepalese study (39%) [15] in Kapilvastu but more than another Nepalese study (14%) [14]. In the former study of Kapilvastu, the overall community had a lower education status and also less access to health education and information. This is in contrast to Chandrashekhar et al. [14] whose population was characterised as one of the most educated (82% as of 2012) and highly accessible urban area of Nepal [26]. This setting is among the top five most developed districts in Nepal [26]. It has two major municipalities, two tertiary hospitals, has driveable roads in most of the places and a good coverage of television and newspapers. Neither study was based on a national data, therefore, due to such differences, the national prevalence of prelacteal feeds is likely to be different from the previous studies. Based on our study one in four children were provided with prelacteal feeds which presents a challenge to achieve the WHO recommended 90% rate of practice of exclusive breastfeeding among under six months children [2].

Economic status and the mother’s education status were significant factors associated with the introduction of prelacteal feeds in this study. This finding is similar to many other studies which show the protective effect that lower economic status has on exclusive breastfeeding [27,28]. The lower socio-economic groups may have less access to the expensive prelacteal feeds such as ghee or honey and therefore exclusive breastfeeding is the only option available to them [27]. This might be a reason for the reported lower prelacteal feeding practice rates amongst the poorest wealth groups in Nepal. There was a protective effect from education up to high school education similar to other studies reported from developing countries [29,30]. However, a notable finding was that the practice of introduction of prelacteal feeds was not significantly different between mothers who did not have formal education and the mothers who had higher education. Further studies need to explore the reason for such higher levels of prelacteal feeds among the highly educated mothers. Nevertheless, this finding indicates that there is need to focus on both of these groups while implementing breastfeeding promotion programs. This is crucial as mothers with higher education are conventionally not a target group of health promotion programs.

The association of ANC visits with birth order, birth interval, ecological region and development region are important findings to consider. Attending the recommended antenatal care visits where exclusive breastfeeding is encouraged should decrease prelacteal feeds and facilitate mothers to get information from the midwives and health workers. Visiting antenatal/maternal and child health (MCH) clinics where they have better access to and exposure to health information and education materials may be the reason that there was a lower prevalence of prelacteal feeds among the mothers who had attended four or more antenatal care visits [31].

First time mothers were more likely to introduce prelacteal feeds in this study. It is likely that the first time mothers have less skill and knowledge of newborn care and proper infant feeding practice [32] and they may rely more on the older women in the household and community who are likely to be less educated than their daughters-in-law and may be more likely to follow the culture and traditions [33].

Regional differences in breastfeeding and newborn care practices could be in part a function of access to service, inequitable distribution of service, information and resources and geographic differences. Mothers from the Far western region of Nepal were less likely to provide prelacteal feeds. The Far western development region of Nepal has some of the poorest areas in the country and is characterised by a harsh terrain with less access to driveable roads, greater reliance on an animal based transport system, and a lower density of the health facilities with the highest child mortality rates [34,35]. The current findings could be a reflection of a large number of mothers from poorest socio-economic groups and a lack of access to expensive prelacteal feeds such as ghee, honey or glucose solution. There is a further need to explore why the prelacteal feeds were introduced to fewer children in the Far western region than other regions of Nepal and how this has influenced the duration of exclusive breastfeeding practice in the region.

The current study found that the mothers from Terai/Plain region were more likely to provide the prelacteal feeds to their newborn. A previous Nepalese study [15] also reported a higher (39%) proportion of mothers introducing prelacteal feeds. The majority of population of the plain/Terai area is similar to the population reported by the study of Kapilvastu district [15]. The majority of the communities in the Terai/Plain area includes a different ethnic group called *Madhesi* (originating from the Plains of Nepal) [36]. This group is generally less educated, have a lower social development status and more likely to follow the traditional practices of prelacteal feeding [36]. A study based in the southern area of Nepal (plains of central development region) reported that despite having all mothers breastfeeding, the initiation of breastfeeding within one hour (3.4%) and the practice of exclusive breastfeeding (72% of the infants were partially breastfed) were poor [36]. Further analysis was not possible as separate record for the *Madhesi* group was not available in the dataset.

### Public health significance of current study

This study further re-iterates the importance of health education and antenatal programs that promote exclusive breastfeeding and discourage the practice of prelacteal feeding. Health institutions and related stakeholders need to examine how they can increase attendance of antenatal care visits which is linked to improved EBF rates. These findings further give strength to the recommendation that all medical and non-medical staff be provided with the adequate information and skill on lactation counseling with a focus on discouraging prelacteal feeds. Female community health volunteers are trained in health promotion and nutrition areas and may be mobilized at the community level for peer counseling to promote exclusive breastfeeding in Nepal [31,32]. In Nepalese society, the decision regarding the utilisation of maternal and child health services and determining infant feeding practice are likely to be made by the mothers-in-law and senior women in the community [37,38]. Mothers-in-law are likely to be less educated and less informed about current breastfeeding practices and therefore, more likely to follow the more traditional practice (including the introduction of prelacteal feeds) than the younger generation. Breastfeeding promotion programs should focus on these senior women and mothers-in-law [25] to improve the uptake of exclusive breastfeeding. The distribution of introduction of prelacteal feeds was not even across Nepal and this study indicates that the Terai/Plain region needs additional attention in implementing breastfeeding promotion programs to improve EBF.

### Strengths and limitation of the study

The nationally representative large sample, an internationally validated study method, and the use of validated questionnaire are major strengths of this study [16]. The application of statistically sound analysis procedures take into account sample weight and cluster design, therefore, the estimation reported in the study is precise. This is the first nationally representative study to report on factors associated with the introduction of prelacteal feeds. Despite many strengths, there are some limitations in the study. First, the study is a cross-sectional study, which limits the ability to draw any causal inferences. Second, the study uses information based on the recall and this increases the likelihood of recall bias. Despite these limitations, the current study does provide representative findings which can be generalisable to the entire country.

## Conclusions

One in four children were provided with prelacteal feeds in Nepal. The prelacteal feeds included milk other than breastmilk, tea, fruit juice, sugar/salt solution, grip water, sugar/glucose solution and plain water. The recommended four antenatal care visits, mother’s education, working status of mother, lower wealth status of family and being from far western development region were protective against the introduction of prelacteal feeds. However, being from the Terai/plain region, being a first time mother and being from the central development region was significantly associated with the introduction of prelacteal feeds. Breastfeeding promotion is essential to promote/encourage the practice of exclusive breastfeeding and reduce the practice of providing prelacteal feeds [39]. The importance of increasing antenatal care visits as a method to increase the practice of exclusive breastfeeding [22,39] should also be noted in future program planning.

## Competing interests

The authors declare that they have no competing interests.

## Authors’ contributions

VK conceived the concept of study design, and performed statistical analysis. VK with significant contribution from MA wrote the first draft of the manuscript. YZ contributed to reanalysis of the data and revising the manuscript. KS contributed to revising the manuscript with significant intellectual contribution. All of the authors contributed to revisions and agreed on the content and views expressed in the manuscript.
